# Seizures and movement disorders induced by hyperglycemia without ketosis in elderly

**Published:** 2014-07-04

**Authors:** Samia Younes, Yousra Cherif, Mouna Aissi, Wafa Alaya, Olfa Berriche, Amel Boughammoura, Mahbouba Frih-Ayed, Baha Zantour, Mohamed Habib Sfar

**Affiliations:** 1 Department of Endocrinology and Internal Medicine, School of Medicine, University of Monastir, Al Munastîr, Tunisia; 2 Department of Neurology, School of Medicine, University of Monastir, Al Munastîr, Tunisia

**Keywords:** Non-Ketotic Hyperglycemia, Hyperglycemia Without Ketosis, Seizures, Movement Disorder, Chorea-Ballismus

## Abstract

**Background: **Non-ketotic hyperglycemia (NKHG) may increase the probability of seizures and movement disorders.

**Methods:** We describe a series of 14 elders admitted for seizures and movement disorders linked to NKHG.

**Results: **Twelve patients developed motor seizures and two others movement disorders. Glucose levels varied 9.28 to 32 mmol/l, while osmolarity values varied from 302.28 to 328 mosmol/l. All patients responded well to insulin therapy and four of them needed anti-epileptic drugs.

**Conclusion: **Seizures or movement disorders in elderly with NKHG could be misdiagnosed as neurological diseases. Blood glucose must be audited whenever patients with seizures or movement disorders are encountered, as the condition may quickly resolve when NKHG is controlled.

## Introduction

Seizures and movement disorders related to non-ketotic hyperglycemia (NKHG) have been reported with increasing frequency since the first case documented in 1965.^1^^,^^2^ The clinical spectrum of this syndrome is various with a severe course in elderly. It develops more quickly than other disorders of diabetes mellitus with hyperglycemia, but usually without evidence of ketosis.^2^^,^^3^ Since the syndrome often ensues during the course of any illness and yet has not been reported in diverse medical fields, doctors must become familiar with this preventable condition, especially in elders. Thus, only the prompt institution of appropriate insulin therapy will improve prognosis and hasten recovery.^2^^,^^3^

## Materials and Methods

We performed a retrospective study of 14 patients referred to the Department of Internal Medicine and Endocrinology over a period of 6 years between 2006 and 2012, on the basis of clinical and biological data consistent with seizures and movement disorders related to NKHG.

Our patients satisfied the inclusion criteria of NKHG: hyperglycemia with no evidence of ketosis, seizures that disappeared when hyperglycemia is controlled and the absence of lesions in cerebral imaging that may explain seizures. Exclusion criteria included a family or personal history of epilepsy, previous cranial trauma, and preexisting neurological disorders associated with seizures.

All patients underwent complete physical examination and routine laboratory analyses, including blood cell count, plasma glucose, serum electrolytes, renal, and liver tests. Urine samples were tested for glucose and ketones. Blood pH was measured in the ward laboratory. Hourly monitoring of the plasma glucose was undertaken at the bedside using dextrostix. Diagnosis of NKHG was based on hyperglycemia, without ketoacidosis and no abnormalities in cerebral imaging and electroencephalogram (EEG).

The appropriate amounts of physiological saline were given. Soluble insulin was injected as soon as the diagnosis was carried. The dose of intramuscular insulin was calculated upon the patient’s weight. The initial dose was 0.1 U/kg hourly until the acute symptoms had been overcome and the plasma glucose had fallen. The medical records of the patients were reviewed for follow-up and response to treatment.

## Results

A total of 14 patients (6 men and 8 women) were included. The main clinical, biological findings, cerebral imaging, EEG, and treatment of the patients are summarized in table 1.

***Clinical details***

The female to male sex ratio was of 1.33. The mean age at the onset of seizures and movement disorders was 75 ± 3 years (71-80 years). The duration of the follow-up ranged from 1 to 31 months (Median of 9 months). The previous medical history of patients was noteworthy with diabetes mellitus in all cases and arterial hypertension in three cases. Four patients experienced partial motor seizures, which were secondarily generalized in only one case. The clinical course was complicated with a partial status epilepticus in another case. Generalized tonic-clonic seizures marked six patients. Movement disorders were present in two cases: a sudden onset of left hemiballism in one case and a left hemichorea in another case.

The patients’ vital signs were stable. The blood pressure was within the normal range in all patients even suffering from arterial hypertension during hospitalization. Eight patients were conscious, and one was obtunded at admission. A thorough neurological examination was normal in 10 cases and revealed a post-critical regressive neurological hemiparesis (Todd’s paralysis) in three patients.

***Paraclinical profiles***

The paraclinical data revealed elevated plasma glucose in all patients. At admission, the plasma glucose levels varied from 9.28 to 32 mmol/l with a mean level of 24.01 mmol/l, but in only five patients the blood creatinine level was over 127 µmol/l. The mean values of serum sodium, serum potassium, and plasma osmolarity were 135.44 mmol/l (132-145 mmol/l), 4.17 mmol/l (3.2-5.0 mmol/l), and 318.88 mosmol/l (302.28-328 mosmol/l), respectively. There was no evidence of ketosis in all urine samples. Blood pH, when performed, was normal or rather elevated in all cases.

The electrocardiogram (ECG) monitoring showed no pronounced peaking of the T-waves at admission. In addition, computed tomography brain was normal in seven patients. In the other cases, it showed cortico-subcortical atrophy (CSCA) in four cases, and ancient lacunar strokes in three other cases. The density of ancient lacunar infract is uniform like the spinal fluid. Cerebral magnetic resonance imaging (MRI) that lacked in 11 cases, revealed a frontal lobe meningioma in case 6 and CSCA, leukoaraiosis and an ancient left pontine stroke in case 8 and CSCA in case 13 (Table 1). The interictal or ictal EEG was recorded in only 10 patients. It was often normal in nine cases or found especially rapid bilateral peaks in the frontotemporal area (Case 3).

***Treatment and follow-up***

Several amounts of physiological saline were infused as a first priority to relieve dehydration at the following rates: 1000-2000 ml in 1 h, 500-1000 ml considering the severity of renal failure. Potassium chloride was added in amount 10 mmol (mEq) per 500 ml of physiological saline as soon as the first dose of insulin was given, provided the ECG showed no peaks of T-waves. All our patients were administered immediately intravenous insulin with a target dextrostix ranged between 7.5 and 10 mmol/l, to which six of them initially responded well. Monitoring of the plasma glucose was undertaken subsequently using dextrostix, and potassium deficiency was controlled every 4 h. The injections were repeated at hourly intervals until the plasma glucose had fallen below 10 mmol/l. When the acute symptoms of seizures and movement disorders relieved within a mean of 2 ± 1 day (1-4 days), intravenous insulin was withdrawn and insulin was injected subcutaneously every 4 h. Anti-epileptic drugs were prescribed during the hospital stay in one case of partial epilepticus status and in three cases of persistent and recurrent seizures despite appropriate insulin therapy.

The clinical course was notable over 3 months with relapses of seizures in cases 2, 4, and 5 (Table 1). In all cases, patients were thereafter referred to the Department of Endocrinology for management of the diabetes mellitus.

## Discussion

Uncontrolled hyperglycemia remains a frequent complication in old patients with diabetes mellitus. The seizures associated with NKHG are seen most often beyond the 5^th^ decade of life^2^^-^^6^ with the mean age of 64 years, albeit few cases have been reported in children.^2^ It tends to present in female patients.^2^^,^^4^^,^^7^ The female to male ratio remains controversial.^2^ The present series is consistent with literature, with a mean age at the onset of 75 years and the female to male sex ratio was 1.33.

All the patients were known as diabetics evolving since about 10 years (5-20 years). The series in the literature showed that in 50% of cases seizures initiated an unknown diabetes.^2^^,^^3^^,^^8^

**Table 1:** Clinical and paraclinical details of 14 elderly patients with seizures and movement disorders related to non-ketotic hyperglycemia on admission and discharge

**Age ** **(years)/sex**	**Duration of ** **MD (years)**	**Seizures and/or ** **movement ** **disorders**	**Physical ** **examination**	**Plasma ** **glucose ** **(mmol/l)**	**Osmolarity ** **(mmol/l)**	**Blood ** **(pH)**	**Plasma ** **Na** ^+^ **/K** ^+^ **(mmol/l)**	**Creatinin** **e (µmol/l)**	**Cerebral imaging**		**EEG on admission/interictal**	**Treatment**	**Symptoms ** **vanishing ** **after (day)**	**Follow-up 3 ** **months**
									**CT**	**MRI**				
76/F	14	PMS in right hemi-body	Memory disturbance	31.80	320	7.54	133/3.2	53	CSCA	-	ND/ND	Insulin	1	No recurrence
77/M	20	GTCS	N	17.70	319	ND	145/3.7	70	N	-	N/ND	Insulin, valproate	1	Recurrent seizures
73/F	7	PMS in right hemi-face	Coma right hemiparesis	20.40	313	ND	132/4.2	69	N	-	ND/fronto-temporal epileptogenic area	Insulin, valproate	1	No recurrence
71/F	5	GTCS→PSE	N	32.00	328	7.47	135/4.1	88	N	-	N/ND	Insulin	4	Recurrent seizures
71/M	7	GTCS	Left hemiparesis	19.05	309	ND	138/3.7	127	LS	-	N/ND	Insulin, clonazepam	3	Recurrent seizures
80/F	15	Right versive	Right hemiparesis	27.00	320	7.44	138/4.1	153	LS	Frontal hygroma	ND/ND	Insulin	2	No recurrence
79/M	5	PMS on right upper limb secondarily GTCS	N	20.85	319	7.42	133/4.5	160	CSCA	-	N/ND	Insulin	1	No recurrence
76/F	12	Left hemiballism	N	28.80	324	7.33	132/5	145	LS	CSCA leukoaraiosis ancient left pontine stroke	ND/ND	Insulin	4	No recurrence
78/F	10	Left hemichorea	N	19.50	318	ND	133/4.5	158	CSCA, leukoaraiosis	-	N/ND	Insulin	4	No recurrence
72/F		PSE	N	14.54	295.34	ND	140/3.8	65	CSCA	-	N/ND	Insulin, Valproate	2	No recurrence
68/F		PMS	N	13.00	305	ND	139/4.1	79	N	-	ND/ND	Insulin	1	No recurrence
72/M		GTCS	N	18.60	323	ND	138/4.2	75	N	-	N/ND	Insulin	3	No recurrence
77/M		GTCS	N	15.08	302.28	ND	135/4.4	82	N	CSCA	N/ND	Insulin	2	No recurrence
75/M		GTCS	N	9.28	315	ND	137/3.7	69	N	-	N/ND	Insulin, Valproate	1	No recurrence

MD: Mellitus diabetes; CT: Computed tomography; MRI: Magnetic resonance imaging; CSCA: Cortico-subcortical atrophy; PMS: Partial motor seizures; GTCS: Generalized tonic-clonic seizures

N: Normal; PSE: Partial status epilepticus, LS: Lacunar stroke, EEG: Electroencephalogram, ND: Not done

The occurrence of seizures may also like all our patients, be a feature of diabetic decompensation in patients with known diabetes.^2^^,^^8^

In fact, a family history of diabetes or the current use of drugs, especially steroids and a previous history of polyuria, polyphagia, and polydypsia should be sought.^3^

Different types of seizures were noticed.^2^^,^^3^ They are motor seizures at the rate of 75-86%,^9^ usually focal,^2^^,^^3^^,^^8^ rarely generalized tonic-clonic seizures. Seizures associated with NKHG are often recurrent, and partial status epilepticus were observed.^3^^,^^7^^,^^9^ Other neurological manifestations are reported in the literature, such lateral hemianopia, visual hallucinations,^2^^,^^9^^-^^11^ cortical blindness,^12^ recurrent giratory seizures^13^ and chorea or hemiballism^3^^-^^6^^,^^10^^,^^14^^-^^22^ or even scarce biballism.^5^^,^^11^^,^^18^ Four patients of the present study experienced partial seizures (Cases 1, 3, 6 and 7), which may secondarily generalized (Only case 7). Generalized tonic-clonic seizures marked six patients. The clinical course of case 4 was complicated with a partial status epilepticus. Movement disorders were present in two cases: hemiballism in case 8 and hemichorea in case 9. The acute symptoms were most often spontaneous,^2^ but they can be also triggered by motion, infection or other factors disturbing diabetes mellitus. No triggering factor was found in our patients.

The physical examination may show severe dehydration. The thorough neurological examination is frequently normal^2^^,^^3^ or may find like in three of our patients a post-critical regressive neurological hemiparesis^2^^,^^3^ (Cases 3, 5, and 6).

Plasma glucose largely exceeds 20 mmol/l.^2^^,^^3^^,^^17^ Not only lower values have been reported,^3^ but also higher values which are associated with severe disorders of consciousness.^2^^,^^17^ The plasma osmolarity is normal or slightly increased.^2^^,^^3^ Serum sodium is normal or low increased; however hyponatremia was described, especially in partial status epilepticus,^2^^,^^9^ unlike our patient. Serum ketosis is absent or present but in trace amounts.^2^ Hyperglycemia ranged from 9.28 to 32 mmol/l in our series. Hyponatremia was found in five of our patients and the serum osmolarity did not exceed 328 mosmol/l. 

The MRI is usually normal,^2^^,^^3^^,^^5^^,^^9^ but it may reveal cerebral abnormalities such as hyperintensity involving the striatum,^5^^,^^10^^,^^15^^,^^19^ the putamen,^4^^,^^18^^,^^19^^,^^23^ the caudate and Globus pallidus^4^^,^^18^^,^^19^ and rarely subthalamic nucleus lesion.^19^^,^^20^ The existence of an abnormality suggests an underlying focal lesion.^2^^,^^8^^,^^17^ In our series, cerebral imaging revealed abnormalities that were not related to the occurrence of seizures or movement disorders. It revealed CSCA in six cases and lacunar strokes in three other cases. Indeed, the abnormality may disappear after stabilization of hyperglycemia and even so the movement disorder or seizures did not resolve.^23^ The EEG may be normal like most of our cases.^2^^,^^9^ It may be a prime investigation especially in visual hallucinations and homonymous hemianopia.^9^^,^^10^ The interictal abnormalities were most often found in the frontal,^2^ more rarely temporal or occipital areas.^9^^,^^10^ The interictal EEG of case 3 showed frontotemporal localized peaks. 

The differential diagnosis of NKHG might be excluded.^18^^,^^22^^,^^23^ First, seizures may occur in diabetic patients as a result of severe renal and hepatic failure, cerebral stroke, drug intoxication or any other etiology in the non-diabetic subject.^13^^,^^18^^,^^23^

The accurate pathogenesis of seizures and movement disorders related to NKHG remains still unclear. Currently, multiple factors, such as vascular lesions, local brain damage, and metabolic factors may contribute to this condition. It partially involves hyperglycemia.^2^^,^^16^ In addition, hyperglycemia increases the metabolism of gamma-aminobutyric acid, and then reduces the threshold of seizures.^2^^,^^4^^,^^9^^,^^14^^-^^16^ Hyponatremia sometimes seems to worsen seizures secondary to NKHG. Singh et al.^7^ showed that partial status epilepticus had been protracted in cases of associated hyponatremia with NKHG. Few studies performed brain spectroscopy and revealed the decrease in N-acetyl aspartate and the brain glucose metabolism^4^^,^^6^^,^^9^^,^^10^^,^^14^^,^^15^^,^^18^^,^^19^^,^^21^^,^^24^ in seizures as far as in chorea. In both brain biopsies of the putamen of two patients showed necrotic foci surrounded by significant gliosis, with prominent infiltration of gemistocytic astrocytes.^4^^,^^10^^,^^13^^,^^21^^,^^25^ Recently, brain needle biopsy has revealed obliterative vasculopathy with prominent vascular proliferation.^3^^,^^14^^,^^25^ Pisani et al. results provide the first evidence that an unrecognized acanthocytosis in diabetes might bring NKHG.^26^

The symptoms are typically refractory to antiepileptic drugs but respond well to insulin therapy.^2^^,^^3^^,^^13^ Nonetheless, it requires sometimes the antiepileptic and neuroleptic drugs in movement disorders.^2^^,^^3^^,^^5^^,^^6^^,^^17^^,^^22^ The antiepileptic drugs especially phenytoin may be harmful and inhibit the insulin secretion.^2^^,^^8^ In our series, the anti-epileptic drugs were prescribed in four patients and have in most cases quickly achieved a satisfactory improvement. Clonazepam was prescribed in emergency settings to treat severe recurrent seizures that may disclose to status epilepticus. When seizures were typically spaced in time, we prescribed the sodium valproate. These antiepileptic drugs were thereafter withdrawn. However, three of patients have recurrent seizures for longer than 3 months, although it is typically much milder than at presentation. Atypical cases have been reported with delayed onset after resolution of the hyperglycemia, unremitting severe disorder movements, and late recurrence.^4^^,^^5^^,^^22^^,^^25^

The prognosis of seizures and movement disorders related to NKHG has been reported to be excellent,^2^^,^^3^^,^^8^^,^^13^^-^^15^ and it was indeed excellent in our all patients too. Nevertheless, Kaseda et al. reported a case of recurrent hemichorea for longer than 1 year in whom they applied 10 daily sessions of low-frequency repetitive transcranial magnetic stimulation over the cortical lesion to reduce the hyperexcitability and get rid of movement disorder.^14^

There are two limitations of our study, first is the limited sample size of elders and secondly, the short follow-up and further implications were not assessed. We did not compare our results to the younger population. Then, at present, we are unable to predict if elderly are more vulnerable to develop NKHG.

## Conclusion

The association of recent onset of seizures with NKHG is striking. Prompt treatment would substantially improve the outcomes of NKHG and avoid the use of antiepileptic drugs and their potential harm in elderly with major comorbidities. Although the appropriate therapy must be started, the search for a focal lesion should be pursued especially in old polymedicated patients. Thereby, we believe that familiarity with seizures and movement disorders related to NKHG will help in questing the differential diagnosis without delay. These cases suggest that acute NKHG should not be delayed as a possible etiology of chorea-ballismus and seizures in elderly, given that diabetes is usually longstanding and poorly controlled and the variable response of elderly diabetics to insulin therapy.

## Notes

**How to cite this article: **Younes S, Cherif Y, Aissi M, Alaya W, Berriche O, Boughammoura A, et al. Seizures and movement disorders induced by hyperglycemia without ketosis in elderly. Iran J Neurol 2014; 13(3): 172-6.
